# TDP2 suppresses genomic instability induced by androgens in the epithelial cells of prostate glands

**DOI:** 10.1111/gtc.12770

**Published:** 2020-05-05

**Authors:** Md. Rasel Al Mahmud, Kenichiro Ishii, Cristina Bernal‐Lozano, Irene Delgado‐Sainz, Masakazu Toi, Shusuke Akamatsu, Manabu Fukumoto, Masatoshi Watanabe, Shunichi Takeda, Felipe Cortés‐Ledesma, Hiroyuki Sasanuma

**Affiliations:** ^1^ Department of Radiation Genetics Graduate School of Medicine Kyoto University Kyoto Japan; ^2^ Department of Oncologic Pathology Mie University Graduate School of Medicine Tsu Japan; ^3^ Centro Andaluz de Biología Molecular y Medicina Regenerativa (CABIMER) CSIC–Universidad de Sevilla Universidad Pablo de Olavide Sevilla Spain; ^4^ Department of Breast Surgery Graduate School of Medicine Kyoto University Kyoto Japan; ^5^ Department of Urology Graduate School of Medicine Kyoto University Kyoto Japan; ^6^ RIKEN Center for Advanced Intelligence Project Tokyo Japan; ^7^ Topology and DNA Breaks Group Spanish National Cancer Research Centre (CNIO) Madrid Spain

**Keywords:** androgen, atypical epithelial hyperplasia, DNA double‐strand break, prostatic intraepithelial neoplasia, TDP2, topoisomerase 2

## Abstract

Androgens stimulate the proliferation of epithelial cells in the prostate by activating topoisomerase 2 (TOP2) and regulating the transcription of target genes. TOP2 resolves the entanglement of genomic DNA by transiently generating double‐strand breaks (DSBs), where TOP2 homodimers covalently bind to 5′ DSB ends, called TOP2‐DNA cleavage complexes (TOP2ccs). When TOP2 fails to rejoin TOP2ccs generating stalled TOP2ccs, tyrosyl DNA phosphodiesterase‐2 (TDP2) removes 5′ TOP2 adducts from stalled TOP2ccs prior to the ligation of the DSBs by nonhomologous end joining (NHEJ), the dominant DSB repair pathway in G_0_/G_1_ phases. We previously showed that estrogens frequently generate stalled TOP2ccs in G_0_/G_1_ phases. Here, we show that physiological concentrations of androgens induce several DSBs in individual human prostate cancer cells during G_1_ phase, and loss of TDP2 causes a five times higher number of androgen‐induced chromosome breaks in mitotic chromosome spreads. Intraperitoneally injected androgens induce several DSBs in individual epithelial cells of the prostate in TDP2‐deficient mice, even at 20 hr postinjection. In conclusion, physiological concentrations of androgens have very strong genotoxicity, most likely by generating stalled TOP2ccs.

## Graphical abstract

The work demonstrates the role of TDP2 for repairing DSBs induced by androgen in human prostate cancer cell lines and epithelial cells of the murine prostate. In addition, increased cellular proliferation rate and cell number have been observed in the absence of functional TDP2 in the prostate. In sum, this work provides mechanistic insight on the interactions between a sex hormone and DNA repair mechanism and the consequence of the perturbation of this interaction.

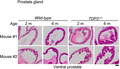

## 1 INTRODUCTION

Sex hormones, estrogens and androgens, strongly stimulate the proliferation of epithelial cells in the mammary glands and prostate, respectively (La Vignera, Condorelli, Russo, Morgia, & Calogero, 2016; Liang & Shang, 2013). Activated estrogen receptors α/β (ERs) and androgen receptor (AR) quickly induce transcription of ER‐ and AR‐responsive genes as transcription factors (Kokontis, Takakura, Hay, & Liao, 1994; Shang, Hu, DiRenzo, Lazar, & Brown, 2000; Wang et al., 2011; Yang et al., 2016). The antagonists against these receptors are widely used as first‐line therapies for breast and prostate cancer patients (Horwich et al., 2013; Musgrove & Sutherland, 2009). There are two major mechanisms for chemical cancerogenesis, the stimulation of cellular proliferation and the induction of mutagenesis (Loeb & Harris, 2008). It has been widely believed that sex hormones enhance oncogenesis through the former mechanism but not the latter (Henderson & Feigelson, 2000). However, recent studies have suggested that androgens can drive oncogenesis by activating topoisomerase II (TOP2), which generates DSBs during its physiological catalysis and can generate chromosome translocation (Gómez‐Herreros et al., 2017; Haffner et al., 2010; Nelson, Haffner, & Yegnasubramanian, 2018). It remains unclear how many breaks are actually generated by the physiological concentration of androgens.

Activated ERs and AR trigger signal‐dependent early transcriptional responses by recruiting TOP2 to their promoter and enhancer segments (Manville et al., 2015; Pommier, Sun, Huang, & Nitiss, 2016). TOP2 has been reported to be involved in RNA polymerase II promoter‐pause release upon physiological signals by androgens, insulin, glucocorticoids, N‐methyl‐d‐aspartate (NMDA), retinoic acid, heat shock and serum (reviewed in refs. Austin et al., 2018; Madabhushi, 2018). TOP2 forms a homodimer and resolves DNA catenanes by catalyzing the transient formation of gated DSBs, which is followed by the enzymatic rejoining of the broken strands through intrinsic intramolecular ligation activity (Gale & Osheroff, 1992; Nitiss, 2009). TOP2 becomes covalently bound to the 5′ DNA end of the transiently formed gated DSBs, generating TOP2‐DNA cleavage complexes (TOP2ccs). The catalysis by TOP2 occasionally becomes “abortive” and remains unsealed, leading to the generation of stalled TOP2ccs (Gómez‐Herreros et al., 2014; Hoa et al., 2016). Thus, the sex hormones enhance carcinogenesis, possibly through an increase in the number of stalled TOP2ccs and generation of mutations. Indeed, we previously showed that physiological concentrations of estrogens frequently generate stalled TOP2ccs in G_0_/G_1_ phases (Sasanuma et al., 2018).

Stalled TOP2ccs are repaired by the two‐step process, the removal of 5′ TOP2 adducts followed by the direct ligation of DSBs by nonhomologous end joining (NHEJ). Tyrosyl DNA phosphodiesterase‐2 (TDP2) is the only known enzyme that is capable of accurately removing 5′ TOP2 adducts from stalled TOP2ccs (Ledesma, Khamisy, Zuma, Osborn, & Caldecott, 2009; Schellenberg et al., 2017). NHEJ requires preceding removal of 5′ TOP2 adducts for its direct ligation of the DSBs in G_0_/G_1_ phase (Gómez‐Herreros et al., 2013). The loss of TDP2 causes attenuated transcriptional responses to androgens in prostate cells, suggesting that exposure to androgens may frequently cause the abortive catalysis of TOP2. However, it remains elusive how many stalled TOP2ccs are actually generated by physiological concentrations of androgens. Another unresolved question is the role played by TDP2 in the prevention of prostatic hyperplasia and oncogenesis. The potential causal relationship between the loss of TDP2 and the oncogenesis is suggested by TCGA database, which shows that homozygous deep deletion of the *TDP2* gene is seen in 0.4% and 0.8% of the cancers arising in the breast and prostate tissues, respectively, but not in other cancer types (Sasanuma et al., 2018). Considering the fact that TDP2 is ubiquitously expressed in the human tissues (Fagerberg et al., 2014; Yue et al., 2014), an unresolved question is why are defects in TDP2 seen specifically in the tissues where cellular proliferation is stimulated by the sex hormones, androgens as well as estrogens.

In this study, we examined the genotoxic effect of androgens on TDP2‐deficient human prostate cancer LNCaP cell line and mouse prostate. Physiological concentrations of androgens induced 4.7 and 16 DSBs in individual *wild‐type* and *TDP2^−/−^* cells at G_0_/G_1_ phase, respectively. This genotoxicity depends on both activated AR and TOP2. These data indicate that androgens have strong genotoxicity, the efficient induction of stalled TOP2ccs in G_1_ phase. We also demonstrate the strong genotoxicity of androgen in the prostate epithelial cells of TDP2‐deficient mice. The loss of TDP2 caused the abnormal proliferation of epithelial cells following three times daily injection of androgens into 2‐month‐old mice and also resulted in progressive prostate hyperplasia in 2‐ and 6‐month‐old mice. We propose that TDP2 suppresses abnormal proliferation of the epithelial cells in the prostate gland by promoting the repair of androgen‐induced DSBs and ensuring proper transcriptional responses to androgens.

## 2 RESULTS

### 2.1 Androgens induce DSBs in serum‐starved LNCaP cells

**FIGURE 1:**
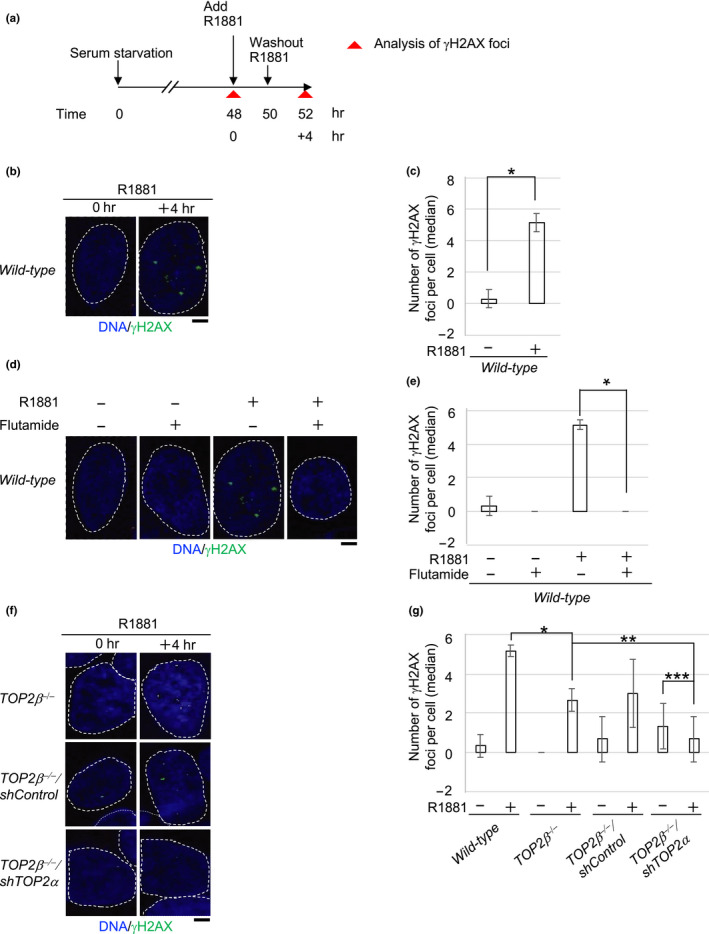
A synthetic androgenic steroid, R1881‐induced DSBs in *wild‐type* LNCaP cells is dependent on both functional AR and TOP2. (a) Schematic diagram of experimental design to examine R1881‐induced γH2AX‐focus formation in human prostate cancer LNCaP cells. After 48‐hr serum starvation, we incubated the *wild‐type* LNCaP cells with 1 nM R1881 for 2 hr, washed‐out R1881, and further incubated the cells without R1881 for 2 hr. γH2AX foci were analyzed at 4 hr after the addition of R1881 (time 52 hr). (b, c) Representative images (b) and median numbers of R1881‐induced γH2AX foci (c). The nuclei are outlined. Error bars show standard deviation (*SD*) of three independent experiments. The number of the counted cells is described in Table S4. A Single asterisk indicates *p* = 2.0 × 10^–4^, which was calculated by an unpaired two‐tailed *t* test. The box plots of γH2AX foci in “c” are shown in Figure S1b. Scale bar represents 25 μm. (d, e) Representative image (d) and median numbers of R1881‐induced γH2AX foci (e). The *wild‐type* LNCaP cells were exposed to R1881 together with flutamide for 2 hr (time 48–50 hr in “a”), followed by incubation in drug‐free media for an additional 2 hr. The nuclei are outlined. Error bars show standard deviation (*SD*) of three independent experiments. The number of the counted cells is described in Table S4. A single asterisk indicates *p* = 6.5 × 10^–6^, which was calculated by an unpaired two‐tailed *t* test. The box plots of γH2AX foci in “e” are shown in Figure S1c. Scale bar represents 25 μm. (f, g) Representative images (f) and median numbers of R1881‐induced γH2AX foci (g) in the indicated genotypes. We analyzed γH2AX foci with the same experimental procedure in “a”. The nuclei are outlined. The generation of the *TOP2β^−/−^*mutant cells is depicted in Figure S1d. Error bars show standard deviation (*SD*) of three independent experiments. The number of the counted cells is described in Table S4. Single, double and triple asterisks indicate *p* = 2.6 × 10^–3^, *p* = 5.5 × 10^–2^ (no significant difference) and *p* = 5.2 × 10^–1^ (no significant difference), respectively, which were calculated by an unpaired two‐tailed *t* test. The box plots of γH2AX foci in “g” are shown in Figure S1g. Scale bar represents 25 μm

To investigate the genotoxic effect of androgens on LNCaP human prostate cancer cells, which express functional AR (Horoszewicz et al., 1983), we enriched G_1_‐phase cells more than 90% by serum starvation for 48 hr (Figure S1a) and analyzed only cyclin‐A‐negative, G_1_‐phase cells. We pulse‐exposed the cells to 1 nM of R1881, a synthetic and orally active androgenic steroid (Tran et al., 2009), for two hours and subsequently removed R1881 from the medium (Figure 1a). The potency of R1881 at 1 nM is comparable to that of 10 nM androgens, a physiological serum concentration after puberty (Corsini et al., 2016; Kemppainen et al., 1999). We then analyzed γH2AX foci 2 hr after the removal of R1881. Remarkably, R1881‐treated LNCaP cells displayed 5.2 ± 0.6 (*SD*; standard deviation) γH2AX foci per cell and 17‐fold induction of γH2AX foci over the baseline (0.3 ± 0.06 (*SD*)) in G_0_/G_1_‐phase LNCaP cells (Figure 1b,c, Figure S1b), indicating the strong genotoxicity of androgens.

### 2.2 Androgen‐induced DNA damage is dependent on a functional androgen receptor

We next investigated whether functional AR is required for androgen‐induced γH2AX‐focus formation in serum‐starved LNCaP cells. We exposed the LNCaP cells to R1881 together with a clinically relevant concentration of an inhibitor against the androgen receptor, flutamide (Schellhammer et al., 1997). The exposure to the antagonist completely repressed R1881‐induced γH2AX‐focus formation in serum‐starved LNCaP cells (Figure 1d,e, Figure S1c). This observation indicates that the activation of the androgen receptor is required for androgen‐dependent DSB formation.

### 2.3 Androgen‐induced DNA damage depends on both TOP2α and TOP2β

We hypothesized that androgen‐induced γH2AX foci were caused by TOP2. There are two isoforms of TOP2, TOP2α and TOP2β (Austin et al., 2018; Madabhushi, 2018), which have an overlapping role in transcription (Sasanuma et al., 2018). To explore this hypothesis, we generated *TOP2β* ^−/−^ LNCaP cells (Figure S1d) and depleted TOP2α more than 20‐fold using shRNA compared with shControl‐treated cells (Figure S1e,f). The loss of TOP2β reduced the number of γH2AX foci at 2 hr from 5.2 ± 0.6 (*SD*) to 2.7 ± 0.6 (Figure 1g, Figure S1g), suggesting that at least 50% of the androgen‐induced γH2AX foci represent stalled TOP2βccs. We previously showed that TOP2α compensates for the lack of TOP2β in the induction of DSBs by estrogens (Sasanuma et al., 2018). We depleted TOP2α by using shRNA and found the resulting *TOP2β* ^−/−^/shTOP2α cells completely suppressed the induction of γH2AX foci by R1881 during G_1_ phase (Figure 1f,g, Figure S1g). These results indicate that androgen‐induced DSBs depend on TOP2α and TOP2β.

### 2.4 Loss of TDP2 causes prolonged γH2AX‐focus accumulation after a pulse exposure of prostate cancer cells to androgens

**FIGURE 2:**
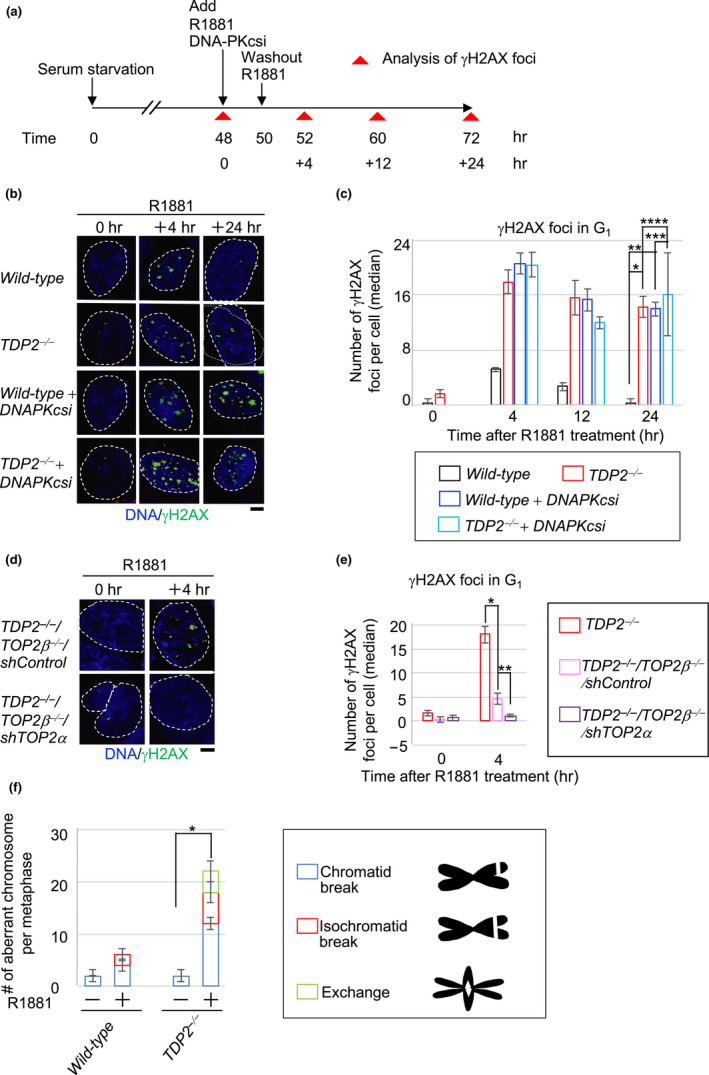
TDP2 functions in the repair of R1881‐induced DSBs in LNCaP cells. (a) Schematic diagram of experimental design to examine the repair kinetics of R1881‐induced γH2AX foci in LNCaP cells. R1881 and DNA‐PKcsi (NU7441) were simultaneously added into the medium at time 48 hr. R1881 was only removed from the medium at time 50 hr, but DNA‐PKcsi exposure was continued until cells were harvested to inactivate NHEJ. γH2AX foci were analyzed at the indicated time (0, +4, +12 and +24 hr after addition of R1881). (b, c) Representative images (b) and median numbers of R1881‐induced γH2AX foci (c) in the indicated genotypes. The generation of *TDP2^−/−^* mutant cells is depicted in Figure S2a. The nuclei are outlined. Error bars show standard deviation (*SD*) of three independent experiments. The number of the counted cells is described in Table S4. Single, double, triple and quadruple asterisks indicate *p* = 1.2 × 10^–4^, *p* = 3.3 × 10^–5^, *p* = 5.7 × 10^–1^ (no significant difference) and *p* = 6.4 × 10^–1^ (no significant difference), respectively, which were calculated by an unpaired two‐tailed *t* test. The box plots of γH2AX foci are shown in Figure S2b. Scale bar represents 25 μm. (d, e) Representative images (d) and median numbers of R1881‐induced γH2AX foci (e) in the indicated genotypes. Error bars show standard deviation (*SD*) of three independent experiments. The nuclei are outlined. The number of the counted cells is described in Table S4. Single and double asterisks indicate *p* = 3.4 × 10^–4^ and *p* = 7.0 × 10^–3^, respectively, which were calculated by an unpaired two‐tailed *t* test. Scale bar represents 25 μm. (f) R1881‐induced chromosome aberrations in mitotic chromosome spread. Following 48‐hr incubation with media containing charcoal‐filtrated serum, cells were further incubated in media containing charcoal‐filtrated serum in the absence (“–”) or presence (“+”) of R1881 (1 nM) for 72 hr. Error bars are standard deviation (*SD*) of three independent analyses. The number of the counted cells is described in Table S4. A Single asterisk indicates *p* = 3.1 × 10^–3^, which were calculated by an unpaired two‐tailed *t* test

To investigate the role of TDP2 in the repair of androgen‐induced DSBs, we disrupted the *TDP2* gene in LNCaP cells (Figure S2a). We pulse‐exposed serum‐starved cells to 1 nM of R1881 for 2 hr and subsequently monitored the resolution kinetics of γH2AX foci at 4 hr, 12 hr and 24 hr after the addition of R1881 (Figure 2a). The pulse exposure to R1881 induced 5.2 ± 0.6 (*SD*) γH2AX foci in *wild‐type* cells and 18 ± 2 (*SD*) γH2AX foci in *TDP2* ^−/−^ cells at 4 hr (Figure 2b,c, Figure S2b). The number of γH2AX foci reduced to a background level in *wild‐type* cells by 24 hr (Figure 2b,c, Figure S2b). In marked contrast, *TDP2^−/−^* cells showed only a ~20% decrease in the number of γH2AX foci from 4 to 24 hr (from 18 ± 2 to 14 ± 2 foci). This result clearly indicates a significant contribution of TDP2 to the repair of androgen‐induced DSBs.

To assess a functional interaction between TDP2 and NHEJ, we inactivated NHEJ by treating serum‐starved *TDP2* ^−/−^ cells with an inhibitor, NU7441 against DNA‐PK‐dependent protein kinase catalytic subunit (DNA‐PKcsi), which enzyme is a central component of NHEJ (Blackford & Jackson, 2017). The inhibition of NHEJ in *wild‐type* cells increased the number of androgen‐induced γH2AX foci from 5.2 ± 0.6 to 21 ± 2 per cell (Figure 2b,c, Figure S2b). *Wild‐type* cells treated with DNA‐PKcsi displayed virtually no decrease in the number of androgen‐induced γH2AX foci from 4 to 24 hr after the addition of androgens (Figure 2b,c, Figure S2b). The data indicate that NHEJ plays an essential role in the repair of androgen‐induced DSBs during the G_1_ phase. Strikingly, the number of γH2AX foci at 24 hr was very similar between *TDP2* ^−/−^ cells and the DNA‐PKcsi‐treated cells (14 ± 2 foci in *TDP2* ^−/−^ versus 14 ± 1 foci in DNA‐PKcsi‐treated *wild‐type* in Figure 2c). Moreover, the loss of TDP2 did not further increase the number of γH2AX foci in DNA‐PKcsi‐treated cells (14 ± 2 foci in *TDP2* ^−/−^ versus 16 ± 6 foci in DNA‐PKcsi‐treated *TDP2* ^−/−^ in Figure 2c). Considering the role of TDP2 in the removal of 5′ TOP2 adducts from stalled TOP2ccs, this epistatic relationship between TDP2 and NHEJ indicates that TDP2 contributes to NHEJ‐mediated repair of androgen‐induced DSBs. In summary, androgens have a very strong genotoxic potential, and collaboration between TDP2 and NHEJ plays a key role in preventing genome instability caused by androgens.

To verify that androgen‐induced DSBs seen in *TDP2* ^−/−^ cells represent stalled TOP2ccs, we created *TDP2* ^−/−^ */TOP2β* ^−/−^/shTOP2α cells (Figure S2c). The inactivation of both TOP2α and TOP2β completely suppressed androgen‐induced DSBs at 4 hr in *TDP2^−/−^* cells (Figure 2d,e). Collectively, TDP2 is required for the repair of TOP2‐dependent DSBs induced by androgens via NHEJ‐mediated repair in G_1_ phase. A physiological concentration of androgens generates ~20 DSBs in individual cells at G_1_ phase, and these DSBs are most likely reflected stalled TOP2ccs.

### 2.5 Androgens induce chromosome breaks in mitotic chromosome spreads

To confirm the genotoxicity of androgens in cycling cells, we quantified the numbers of aberrant chromosomes in mitotic spreads following 69‐hr continuous exposure of cycling cells to 1 nM R1881. R1881 treatment increased chromosome aberrations by 3.0‐fold (from 2.0 ± 0.4 to 6.0 ± 0.8 foci) in *wild‐type* and 11‐fold (from 2.0 ± 1 to 22 ± 2 foci) in *TDP2^−/−^* cells, respectively (Figure 2f). In conclusion, a physiological concentration of androgens produces a few aberrant chromosomes even in *wild‐type* cells, and TDP2 plays an important role in the faithful repair of androgen‐induced chromosome aberrations.

### 2.6 Androgens induce prominent γH2AX foci in prostate epithelial cells of *TDP2* ^−/−^ mice

**FIGURE 3:**
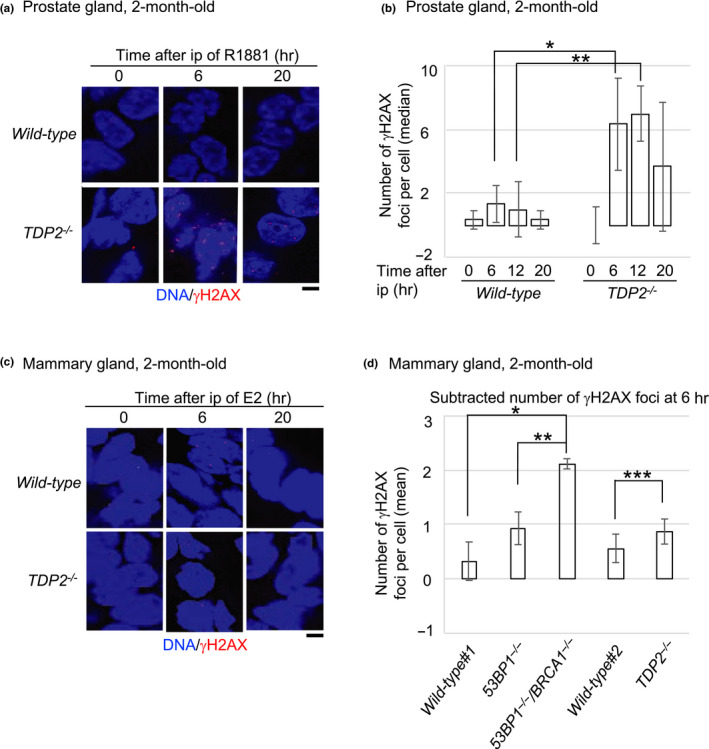
Genotoxicity of R1881 and 17β‐estradiol in the prostate and mammary tissues, respectively, of *TDP2^−/−^* mice. (a, b) Representative images (a) and median numbers of γH2AX‐focus‐positive (b) cells in luminal epithelial cells of the prostate at the indicated time after ip with R1881. We killed three 2‐month‐old mice of each genotype and counted only luminal cells stained with cytokeratin‐8/18 (CK‐8/18), a marker of epithelial cells. The box plot of R1881‐induced γH2AX foci is shown in Figure S4a. The number of the counted cells is described in Table S4. Single and double asterisks indicate *p* = 4.9 × 10^–2^ and *p* = 1.3 × 10^–2^, which were calculated by an unpaired two‐tailed *t* test. Scale bar represents 25 μm. (c, d) Representative images (c) and the average number of γH2AX foci (d) cells in the epithelial cells of the mammary gland at the indicated time after ip with 17β‐estradiol (E2). We subtracted the average number of foci in E2‐treated epithelial cells at 6 hr from the average number of foci in E2‐untreated epithelial cells. Actual numbers of foci per cell are shown in Figure S4d. The data of *wild‐type* #1, *53BP1^−/−^* and *53BP1^−/−^*/*BRCA1^−/−^* mice were re‐calculated as the number of γH2AX foci per cell from the data that has been previously published (Sasanuma et al., 2018). We counted only cells stained with cytokeratin‐8/18 (CK‐8/18). We killed three mice each carrying either the *wild‐type* #2 or *TDP2* ^−/−^ genotype at two months of age and analyzed more than 100 epithelial cells for each mouse. The number of the counted cells is described in Table S4. Single, double and triple asterisks indicate *p* = 1.0 × 10^–3^, *p* = 1.1 × 10^–2^ and *p* = 2.0 × 10^–1^ (no significant difference), respectively, which were calculated by an unpaired two‐tailed *t* test. Scale bar represents 25 μm

To verify the genotoxicity of androgens in vivo, we administrated R1881 by intraperitoneal injection (ip) into *wild‐type* and *TDP2^−/−^* mice at 2 months of age and monitored the number of γH2AX foci in the prostate, where the expression level of AR is relatively higher than other tissues in both humans and mouse (Figure S3a,b) (El‐Alfy et al., 1999; Mirosevich et al., 1999). The selective expression of AR is detected in the prostate epithelial cells, which highly express cytokeratin 8/18 (Ishii, Imanaka‐Yoshida, Yoshida, & Sugimura, 2008; Wang, Hayward, Cao, Thayer, & Cunha, 2001). R1881 injection caused 1.3 ± 1 and 6.3 ± 3 γH2AX foci per cell at 6 hr after ip in the prostate epithelial cells (cytokeratin 8/18‐positive) of *wild‐type* and *TDP2^−/−^* mice, respectively (Figure 3a,b, Figure S4a) (Toivanen & Shen, 2017). Remarkably, *TDP2^−/−^* mice still displayed 7.0 ± 2 γH2AX foci at 12 hr after ip, while *wild‐type* mice displayed a background level of foci at 12 hr (Figure 3a,b, Figure S4a). We also analyzed the spleen, which poorly expresses AR in both humans and mice (Figure S3a,b) (Fagerberg et al., 2014; Yue et al., 2014). As expected, essentially no γH2AX foci were induced by androgens in the spleen of *wild‐type* or *TDP2^−/−^* mice (Figure S4b,c). Taken together, androgens are highly genotoxic in both human prostate cancer cells and the epithelial cells of the mouse prostate particularly in the absence of TDP2.

We previously reported that intraperitoneal injection of estrogens (17β‐estradiol) induces a several times higher numbers of γH2AX foci at 6 hr after ip in the epithelial cells of the mammary glands in BRCA1‐deficient mice in comparison with *wild‐type* controls (Sasanuma et al., 2018). In this study, we injected estrogens into *wild‐type* and *TDP2^−/−^* mice. The number of γH2AX foci in mammary epithelial cells was only slightly higher in *TDP2^−/−^* mice than *wild‐type* at 6 hr after ip (Figure 3c,d). This result indicates that TDP2 does not play a very important role in the repair of estrogen‐induced DSBs in the mouse mammary gland.

### 2.7 Androgens stimulate the proliferation of prostate epithelial cells in *TDP2^−/−^* mice to a greater extent than in *wild‐type* mice

**FIGURE 4:**
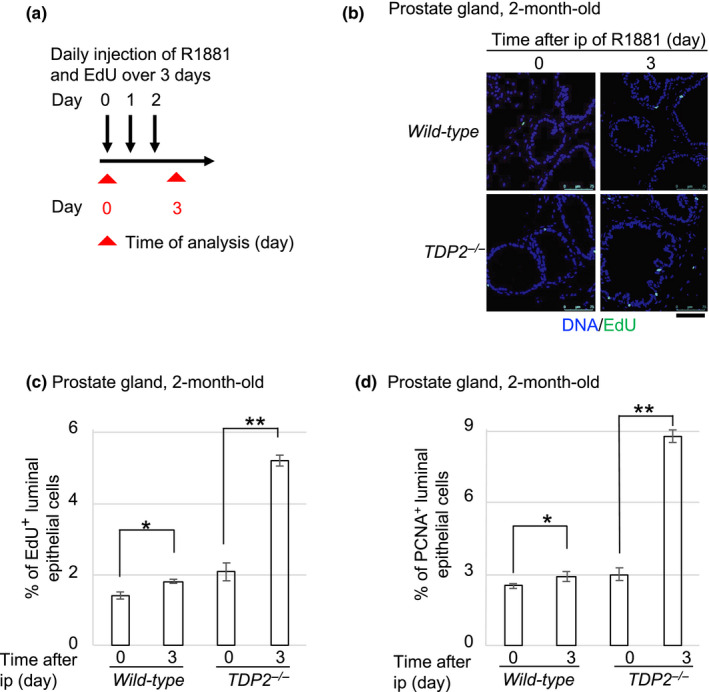
R1881 facilitates the cellular proliferation in the prostate gland of *TDP2^−/−^* mice. (a) Schematic diagram of experimental design for a daily injection of R1881 in 2‐month‐old mice. (b, c) R1881‐induced cellular proliferation by EdU incorporation in the mouse prostate. Representative images (b) of the EdU‐positive (EdU^+^) cells and the average numbers of R1881‐induced EdU^+^ (c) luminal epithelial (CK‐8/18‐positive) cells in the prostate gland of mice. The number of the counted cells is described in Table S4. EdU (30 mg/kg body weight) and R1881 (15 mg/kg body weight) together were intraperitoneally (ip) injected into the mice (days 0, 1 and 2 in “a”). The indicated tissues were isolated 24 hr after the last ip (day 3 in “a”). Small intestine and spleen were analyzed as positive controls in Figure S5a. Single and double asterisks indicate *p* = 2.9 × 10^–3^ and *p* = 5.3 × 10^–5^, respectively, which were calculated by an unpaired two‐tailed *t* test. Scale bar represents 75 μm. (d) R1881‐induced cellular proliferation by PCNA immunostaining in the mouse prostate. The graph shows the average numbers of R1881‐induced PCNA‐positive (PCNA^+^) cells. We analyzed PCNA^+^ cells with the same experimental procedure in “a”. The number of the counted cells is described in Table S4 Single and double asterisks indicate *p* = 3.1 × 10^–2^ and *p* = 1.1 × 10^–5^, respectively, which were calculated by an unpaired two‐tailed *t* test. Representative images of PCNA^+^ cells in the prostate are shown in Figures S5b

Activated AR stimulates cellular proliferation by controlling transcription of AR‐target genes (Wang et al., 2009). A recent study indicates the role for TDP2 in ensuring proper transcriptional response to androgens. We here examined the effect of androgens on the proliferation of the epithelial cells in the mouse ventral prostates, which are very sensitive to androgens (Kerr & Searle, 1973; Sandford, Searle, & Kerr, 1984; Sugimura, Cunha, & Donjacour, 1986), of 2‐month‐old mice. To this end, we daily injected R1881 for three days and examined the prostate at day 4 (Figure 4a). To visualize cycling cells, we injected R1881 together with 5‐ethynyl‐2'‐deoxyuridine (EdU), which is incorporated into newly synthesized DNA during S phase. As expected, substantial numbers of EdU‐positive (EdU^+^) cells were detectable in the epithelial cells of the intestine and splenocytes (Figure S5a) due to the rapid turnover of these cells (CREAMER, Shorter, & Bamforth, 1961; Gelberg, 2007; Kamath et al., 2000). The 3 days’ injection of R1881 showed only a subtle but significant increase in the proliferation of the epithelial cells in *wild‐type* mice (from 1.4 ± 0.1 at day 0 to 1.8 ± 0.1 at day 3) (Figure 4b,c). In contrast, this injection increased the percentage of EdU^+^ epithelial cells by three times in *TDP2^−/−^* mice (from 2.1 ± 0.3 at day 0 to 5.2 ± 0.2 on day 3). Likewise, the 3 days’ injection of R1881 dramatically increased the percentage of the cytokeratin 8/18‐positive epithelial cells (Figure S5b) that expressed the proliferating cell nuclear antigen (PCNA), a conventional biomarker for cycling cells, from 3.0 ± 0.3% to 8.8 ± 0.3% in *TDP2^−/−^* mice, compared with those of *wild‐type* mice (Figure 4d). These results consistently indicate that TDP2 prevents the abnormal proliferation of prostate epithelial cells in response to injected R1881. Considering the role of TDP2 in the repair of stalled TOP2ccs, a defect in their rejoining might change transcriptional responses to androgens and abnormally stimulate the proliferation of the epithelial cells.

### 2.8 Development of hyperplasia of prostate epithelial cells in *TDP2^−/−^* mice

**FIGURE 5:**
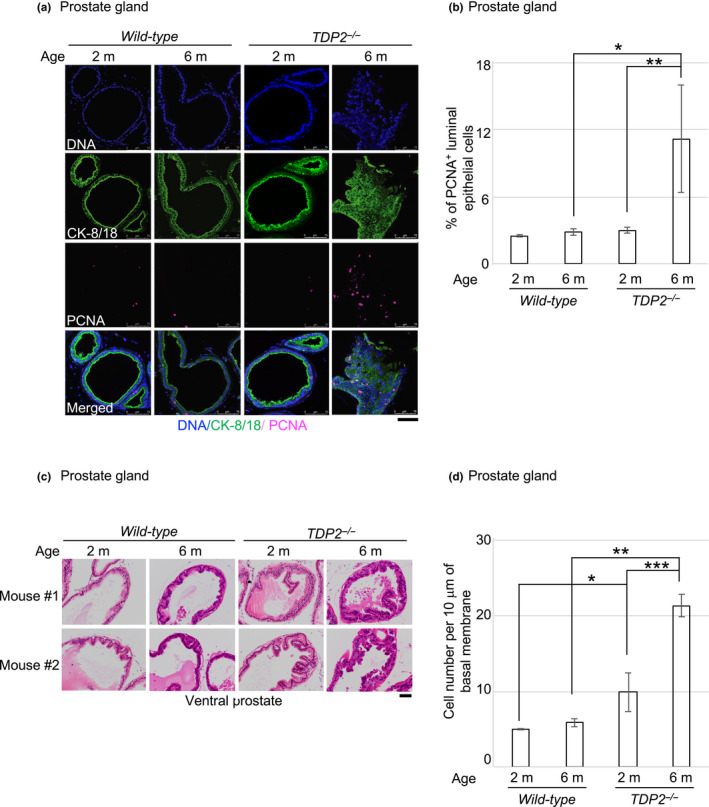
Hyperplasia of ventral prostate in *TDP2^−/−^* mice. (a, b) Spontaneous cellular proliferation by PCNA immunostaining in the mouse prostate. Representative images (a) and the average numbers (b) of PCNA‐positive (PCNA^+^) luminal epithelial (CK‐8/18^+^) cells in the ventral prostate in the indicated genotypes and age‐groups of mice. We killed three 2‐month‐old (2 m) and three 6‐month‐old (6 m) mice of the indicated genotypes. We analyzed PCNA^+^ cells with the same in “a”. The number of the counted cells is described in Table S4. Single and double asterisks indicate *p* = 4.7 × 10^–3^ and *p* = 2.9 × 10^–2^, respectively, which were calculated by an unpaired two‐tailed *t* test. Scale bar represents 75 μm. (c, d) Spontaneously arising hyperplasia of the ventral prostate stained by hematoxylin and eosin (H&E). We analyzed three 2‐month‐old mice and five 6‐month‐old mice of the indicated genotypes. Representative images in “c” from two‐independent experiments were taken with ×200 magnification. The number of epithelial cells in “d” was counted along 10‐μm basement membrane in individual ducts (10 ducts were counted from each mouse) of the ventral prostate in the indicated genotypes and age‐groups of mice. The number of the counted cells is described in Table S4. Error bars show standard deviation (*SD*) calculated by the cell numbers which were counted from at least three mice. The single, double and triple asterisks indicate *p* = 2.9 × 10^–2^, *p* = 2.0 × 10^–8^ and *p* = 1.8 × 10^–4^, respectively, which were calculated by an unpaired two‐tailed *t* test. Scale bar represents 100 μm

The above data indicated that androgens induced several DSBs in individual prostate epithelial cells and strongly stimulated their proliferation in the absence of TDP2. To evaluate the proliferation rate of the epithelial cells, we stained PCNA in the ventral prostate, comparing between 2‐month and 6‐month‐old mice (Figure 5a). The percentage of PCNA^+^ epithelial cells was 2.5 ± 0.1% and 3.0 ± 0.3% in *wild‐type* and *TDP2^−/−^* mice at the age of 2 months, respectively. The percentage did not increase in *wild‐type* mice (2.8 ± 0.3%), while the percentage increased to 11 ± 5% in *TDP2^−/−^* mice at the age of 6 months. One possible scenario is that the accumulation of mutations with aging in the absence of TDP2 might increase the sensitivity of the prostate epithelial cells to androgens leading to their enhanced proliferation (Figure 5b).

We next investigated the consequence of the enhanced proliferation, the hyperplasia of epithelial cells in the prostate. We analyzed the ventral prostate of *wild‐type *and *TDP2^−/−^* mice at 2 months and 6 months of age. We counted the number of epithelial cells along the fixed distance of the lumen surface in 10 ducts of each mouse. *Wild‐type* mice did not exhibit morphological abnormality of ventral prostate (Figure 5c). In contrast, *TDP2^−/−^* mice at both 2 and 6 months of age exhibited hyperplasia in epithelial cells of ventral prostate. The number of epithelial cells was twofold higher in 2‐month‐old *TDP2^−/−^* mice compared with 2‐month‐old *wild‐type* mice (Figure 5c,d). The hyperplasia of the ventral prostate was twofold more frequently observed in 6‐month‐old *TDP2^−/−^* mice than 2‐month‐old ones. Epithelial layers were folded as a consequence of the increase in the number of epithelial cells. Interestingly, 6‐month‐old *TDP2^−/−^* mice exhibited the accumulation of epithelial cells having a disordered, multilayered organization in more than 75% of the examined ducts. Nonetheless, we did not detect malignant tumors. Collectively, TDP2 prevents the abnormal proliferation of epithelial cells in the ventral prostate.

## 3 DISCUSSION

We here reveal previously unappreciated very strong genotoxicity of a physiological concentration of androgens equivalent to a serum concentration in males after puberty. A 2‐hr pulse exposure to androgens induced approximately five TOP2‐dependent DSBs per cell in the G_1_ phase in an AR‐receptor‐dependent manner. The loss of either NHEJ or TDP2 caused an increase in androgen‐induced DSBs to ~20 per cell. Strikingly, this loss resulted in 14 to 16 DSBs left unrepaired even at 24 hr after a 2‐hr pulse exposure to androgens, which is in marked contrast with the complete rejoining of androgen‐induced DSBs in *wild‐type* cells at 24 hr (Figure 2c). A few androgen‐induced DSBs were left unrepaired in individual prostate epithelial cells of TDP2‐deficient mice even at 20 hr after the injection of androgen when androgen‐induced DSBs were completely repaired in *wild‐type* mice (Figure 3b). These data highlight the key role in the TDP2‐NHEJ axis (Gómez‐Herreros et al., 2013) in protecting prostate epithelial cells from androgen‐induced genome instability.

The genotoxicity of androgens as well as estrogens can be mediated by the following two mechanisms. A physiological concentration (1 nM) of 17β‐estradiol generates DSBs in *wild‐type* MCF‐7 breast cancer cells by generating stable RNA:DNA hybrid structures known as R‐loops and their collision with DNA replication forks causes DSBs (Stork et al., 2016). Hence, androgens can generate DSBs through the collision between R‐loops and DNA replication forks. Since R‐loops are not a serious threat to genome instability in G_0_/G_1_ phases (reviewed in ref. Aguilera & García‐Muse, 2012), androgen‐induced DSBs in G_0_/G_1_ phases are generated by a mechanism other than R‐loops. Recent studies have shown the second mechanism for androgen‐induced DSBs, TOP2‐dependent DSBs (Haffner et al., 2010) (reviewed in refs. Madabhushi, 2018; Nelson et al., 2018). We here showed TDP2 plays a vital role in the efficient repair of androgen‐induced DSBs (Figures 2e and 3b), suggesting that they include 5′ TOP2 adducts at their ends. Indeed, TOP2 is essential for the induction of DSBs by androgen in both *wild‐type* and *TDP2^−/−^* cells (Figures 1f and 2e). We, therefore, conclude that a physiological concentration of androgens has very strong genotoxicity by generating stalled TOP2ccs.

We investigated the biological consequence of androgen‐induced DSBs by comparing *wild‐type* and *TDP2^−/−^* mice. There are two alternative hypotheses concerning the effect of TOP2‐dependent DSBs on the signal‐dependent early transcriptional responses in *wild‐type* cells. The first hypothesis assumes that TOP2‐dependent DSBs are programmed and have a physiological role in transcription regulation (reviewed in ref. Puc, Aggarwal, & Rosenfeld, 2017). Alternatively, TOP2‐dependent DSBs are generated by nonphysiological abortive catalysis of TOP2, which catalysis is a stochastic event (Hoa et al., 2016) (reviewed in ref. Morimoto et al., 2019). While the role of androgen‐induced stalled TOP2ccs in *wild‐type* cells remains controversial, previous reports and the current study have consistently shown the pathological effect of androgen‐induced stalled TOP2ccs when TDP2 is absent. Previous reports demonstrate that the loss of TDP2 causes altered transcriptional response to androgens in prostate cancer cells (Gómez‐Herreros et al., 2014). We here showed that *wild‐type* and *TDP2^−/−^* mice displayed 1.3‐fold and threefold increases, respectively, in the proliferation of prostate epithelial cells in response to injected androgens (Figure 4c,d). This observation is reminiscent of the abnormal proliferation of mammary epithelial cells in the absence of functional NHEJ upon the daily injection of estrogen into mice (Itou et al., 2020). Collectively, TDP2 plays a vital role in preventing the pathological accumulation of androgen‐induced stalled TOP2ccs and ensuring appropriate signal‐dependent early transcriptional responses in the control of cellular proliferation. Our data suggest that TDP2 can suppress the oncogenesis of prostate epithelial cells by both preventing androgen‐induced pathological DSBs and ensuring appropriate cellular proliferation in response to androgens.

We showed that the 6‐month‐old TDP2‐deficient mice exhibited ~4 times increase in the proliferation rate of prostate epithelial cells over 6‐month‐old *wild‐type* mice (Figure 5b) and the hyperplasia of epithelial cells (Figure 5d). This observation is in contrast with the data that 2‐month and 6‐month‐old *wild‐type* mice exhibited similar proliferation rates and normal morphology in the epithelial cells. There are two potential mechanisms for the four times increase in the proliferation rate. First, the dysregulation of transcriptional responses to the endogenous androgen may cause abnormal proliferation. Another mechanism is that the genome instability in TDP2‐deficient epithelial cells leads to the accumulation of mutations such as genome rearrangements caused by the misrepair of TOP2‐induced DSBs. We have failed to detect malignant prostate tumors in the TDP2‐deficient mice, presumably due to the presence of the tumor suppressor gene, such as P53 and PTEN (Itou et al., 2020; Jamaspishvili et al., 2018). Inactivation of P53 or PTEN by deletion/mutation is identified in ∼20% of primary prostate tumor samples (Weinstein et al., 2013). Nonetheless, these mice developed at 6 months of age the prominent pathological lesions, the accumulation of abnormal epithelial cells having a disordered and multilayered organization (Figure 5c,d). These lesions are very similar to prostatic intraepithelial neoplasia (PIN), which is believed to precede the onset of prostate cancer within a decade (Brawer, 2005; Cheng, MacLennan, & Bostwick, 2020) according to several prospective studies on prostate carcinogenesis. Moreover, the loss of TDP2 resulted in both ~4 times increase in the proliferation rate (Figure 5b) and a few times increase in the number of epithelial cells (Figure 5d) at the age of 6 months. Taken together, the prominent pathological lesions and the significant increase in the number of proliferating epithelial cells indicate that TDP2‐deficient mice may provide a new model system to explore the role of TDP2 in the prevention of oncogenic transformation and tumor initiation in the prostate.

The present study suggests the differential contribution of TDP2 to genome stability between the mammary gland and prostate. We have previously demonstrated that collaboration between breast cancer susceptibility gene I (BRCA1) and the MRE11 endonuclease promotes the removal of 5′ TOP2 adducts from DSB ends and significantly contributes to the NHEJ‐mediated removal of stalled TOP2ccs independent of TDP2 (Hoa et al., 2016; Sasanuma et al., 2018). The loss of BRCA1 caused a more significant increase in the number of estrogen‐induced DSBs in murine mammary epithelial cells (Figure 3d, Figure S4d; Sasanuma et al., 2018) in comparison with the loss of TDP2 (Figure 3d). Moreover, the loss of TDP2 had only a modest effect on estrogen‐induced DSBs in mammary epithelial cells (Figure 3d) but had a significant effect on androgen‐induced prostate epithelial cells in mice (Figure 3b). Thus, TDP2 plays the dominant role in the removal of stalled TOP2ccs in the prostate may have a marginal role in the mammary glands, at least in mice. A substantial amount of TDP2 is expressed in murine mammary glands (Figure S3d) though the expression of TDP2, specifically in epithelial cells, has not yet been measured. Future studies need to address the relative usage of BRCA1‐MRE11 and TDP2 in the human mammary gland and prostate. The differential relative usage of these enzymes can explain why homozygous long deletion of TDP2 gene is seen more frequently in the prostate cancer than breast cancer (TCGA database) (Sasanuma et al., 2018), while defects in BRCA1 increase the incidence of the breast cancer to a greater extent than that of the prostate cancer (Roy, Chun, & Powell, 2012).

## 4 EXPERIMENTAL PROCEDURES

### 4.1 Cell culture and reagents

Human LNCaP cells were incubated in RPMI1640 medium (Cat# 3026456, Nacalai Tesque) supplemented with horse serum (10%, Gibco), penicillin (100 U/ml) and streptomycin (100 µg/ml, Nacalai). For G_1_/G_0_ arrest by serum starvation, LNCaP cells were incubated in serum‐free medium for 48 hr. LNCaP cells were exposed to DNA‐PK catalytic subunit inhibitor (DNA‐PKcsi), NU7441 at 2 µM concentration in DMSO before lipofection to decrease the NHEJ efficiency and therefore to increase the knockout efficiency.

### 4.2 CRISPR/Cas9‐mediated genome editing in human LNCaP cells

The gRNAs (5′‐TCTGTCAGAGAGGGCTCGAG, and 5′‐ CCAAGAAGGTCCAAACTTCG) were individually inserted into the *Bbs*I site of pX459 (Cat# 48139, Addgene). pX459 expresses gRNA under the control of the U6 promoter and Cas9 under the chicken β‐actin promoter. Schematic diagram of *TDP2* target location is depicted in Figure S2a. pX459‐gRNAs was transfected into LNCaP cells with Fugene HD (Cat#E2311, Promega). Following the transfection, cells were incubated with the puromycin‐containing medium for 40 hr. Afterward, we removed puromycin and further incubated the cells for approximately 3 weeks to isolate the clones. The gene‐disruption events were confirmed by Western blotting analysis.

### 4.3 Lentivirus‐mediated depletion of TOP2α by shRNA

Lentiviral vectors were simultaneously transfected with the virus packaging plasmids (pMD2.G, pMDLg/pRRE and pRSV‐Rev) into LentiX‐293T cells (Cat# 632180, TAKARA). Lentiviruses were harvested at 48 hr post‐transfection. LNCaP cells were infected with the virus for 48 hr. Puromycin was used as the selection marker to enrich the infected cells at 24 hr after infection. For gene silencing, shRNA sequence of TOP2α (5′‐ CCCATTGTAAAGGTATCTAAA) was cloned into a pLKO.1 lentiviral vector. The down‐regulation of TOP2α was confirmed by Western blotting analysis (Figure S1g).

### 4.4 Administration of estrogen and androgen into mice

For a *wild‐type* strain relevant to *TDP2^−/−^*, the C57BL6/J strain was used. We injected E2 (300 µg/kg body weight) and R1881 (300 µg/kg body weight) into *TDP2^−/−^* mice via intraperitoneal injection (ip) at 100 µL volume. The same volume of an E2 and R1881 solvent (PBS) was injected into the control, C57BL6/J mice. We daily administered R1881 for the indicated periods, as shown in Figure 4.

### 4.5 Generation of *TDP2^−/−^* mice and animal maintenance

Generation of *TDP2^−/−^* mice has been described in Gómez‐Herreros et al. (2013). The mouse colony was maintained in an outbred C57BL/6 background under standard housing conditions (21 ± 1°C) with artificial light (12:12‐hr light: dark cycle, lights on at 8 a.m.). Mice were kept in isolated cages. To maintain a specific pathogen‐free environment in the cages,  controlled ventilation was performed using HEPA‐filters.

### 4.6 Ethics statement

The experimental protocol was approved by the Ethical Committee for Animal Experimentation of the University of Seville.

### 4.7 Preparation of thin‐slice specimen from isolated mammary and prostate glands

Prostate gland tissue is harvested from 2‐month‐old and 6‐month‐old male mice. Mammary gland tissue in Figure S4 is harvested from 2‐month‐old‐female mice. To prepare the frozen block for hormone‐induced genotoxicity analysis, the isolated tissues were briefly washed with cold PBS, fixed with paraformaldehyde (4%, Cat# 163‐20145, Wako) for 15 min at 4°C and then washed 3 times with PBS. The tissue samples were incubated with 30% sucrose in PBS for 30 min to 3 hr and then embedded with optimal cutting temperature (OCT) compound (Cat# 4583, Sakura) into Cryomolds (Cat# 4565, Sakura, Japan). The tissues were frozen in liquid nitrogen and kept at −80°C until use. Prior to use, the frozen blocks of the tissues were placed on a cryostat (CM1850, Leica) at −25°C and sectioned into 10‐µm slices. Slides were heated at 55°C for 30 min to dry. To prepare paraffin block for proliferation index and histological analysis, organs were fixed in 4% paraformaldehyde (Cat# 163‐20145, Wako) for overnight, embedded in paraffin and cut into 5‐µm slices by microtome. To analyze proliferation index, paraffin slides were stained with PCNA or labeled with EdU. Slides were stained with hematoxylin–eosin for histopathological analysis and visualized under the microscope. To quantify the number of luminal cells in the ventral prostate (Figure 5d), we measured the length of the basal layer of ventral prostate and counted nuclei by ImageJ software. We then calculated the cell number per unit length (10 µm).

### 4.8 Immunostaining of tissues and cells

Tissue samples were incubated with paraformaldehyde (4%) for 10 min and then washed three times with PBS containing Tween‐20 (0.05%, PBS‐T). The slides were incubated with blocking solution (1% BSA and 5%, goat serum in PBS‐T) for 1 hr at room temperature (or overnight at 4°C) and then washed once with PBS‐T. Slides were then incubated with both α‐cytokeratin‐8/18 (1/10, rat monoclonal, University of Iowa, US) and α‐γH2AX antibody (1/500, 20E3, rabbit monoclonal, Cell Signaling Technologies) for 1 hr (or overnight) at 4°C. After washing with PBS‐T, slides were incubated with both α‐rabbit (Alexa Fluor 546) and α‐rat (Alexa Fluor 488) secondary antibodies (Molecular probe, US). γH2AX and cytokeratin‐8/18 signals were detected using BZ‐9000 (KEYENCE) and LEICA SP8.

LNCaP cell was fixed with ice‐cold methanol for 20 min on ice and permeabilized with 0.5% Triton X‐100 in PBS (0.05% PBS‐T) on ice. After incubation in blocking solution (5%, BSA), cells were incubated with the following antibodies for 1 hr: α‐γH2AX (1/1000, JBW301, mouse monoclonal, Millipore), α‐γH2AX (1/500, 20E3, rabbit monoclonal, CST, US) and α‐Cyclin A (1/500, rabbit polyclonal, ab87359).

### 4.9 Chromosome aberration analysis in mitotic chromosome spreads

R1881‐induced chromosome aberrations in mitotic chromosome spread of LNCaP Cell. Following 48‐hr incubation with media containing charcoal‐filtrated serum, cells were further incubated in media containing charcoal‐filtrated serum in the absence (“–”) or presence (“+”) of R1881 (1 nM) for 69 hr. To enrich metaphase cells, we incubated the cells with the medium containing colcemid (0.1 μg/ml, Thermo Fisher) for three hours and then immediately performed chromosome analysis. Cells were suspended in potassium chloride (75 mM) for 15 min, washed with Carnoy's solution (a 3:1 mixture of methanol and acetic acid), dropped on slides and stained with a Giemsa solution (5%) for 10 min.

### 4.10 Western blot analysis

Cells (1 × 10^6^) were lysed in 100 µl sodium dodecyl sulfate (SDS) buffer, containing Tris‐HCl (25 mM, pH 6.5), SDS (1%), β‐mercaptoethanol (0.24 mM), bromophenol blue (0.1%) and glycerol (5%). Whole‐cell extracts were separated by electrophoresis, transferred onto polyvinylidene difluoride membranes and blocked in skimmed milk (5%) dissolved in Tween‐20 (0.1%) in TBS (TBST). The membranes were incubated with primary antibodies overnight at 4°C followed by washing three times with TBST. The membranes were then incubated with appropriate HRP‐linked secondary antibodies at room temperature for 1 hr and washed thrice prior to signal detection. Membranes were developed by chemiluminescence using ECL reagent.

### 4.11 Focus counting and statistical analysis

Foci images were captured using a BZ‐9000 fluorescence microscope (Keyence). The number of the nuclear foci signals was automatically counted using Hybrid cell count software (Keyence). *p*‐values are calculated by an unpaired Student's *t* test. Error bars represent standard deviation (*SD*), as indicated in the legends. All box plots in the supplementary figures are generated using GraphPad Prism 8.0.

## AUTHOR CONTRIBUTIONS

F.C.‐L. and H.S. as well as S.T. conceived the study. Experiments and data analysis were performed by R.A.M. Paper was written by F.C.‐L., S.T. and H.S.

## Supporting information

Fig S1‐S5

Table S1‐S4
