# Supplementary material for: TDP2 suppresses genomic instability induced by androgens in the epithelial cells of prostate glands
Source: Genes Cells. 2020 May 5;25(7):450–65. doi: 10.1111/gtc.12770 (PMC7497232; doi:10.1111/gtc.12770)

Rasel, *et al.*, Figure S1

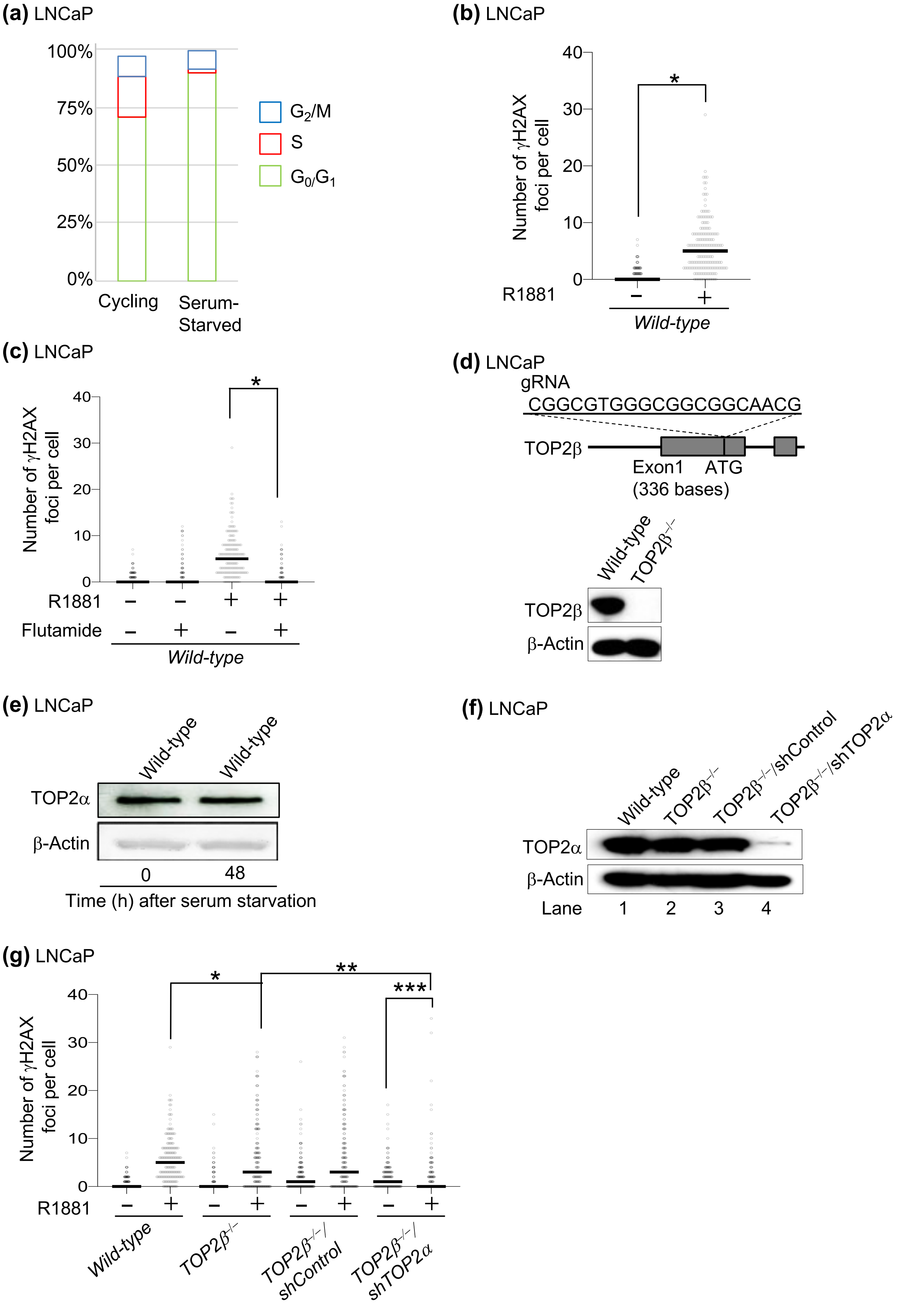

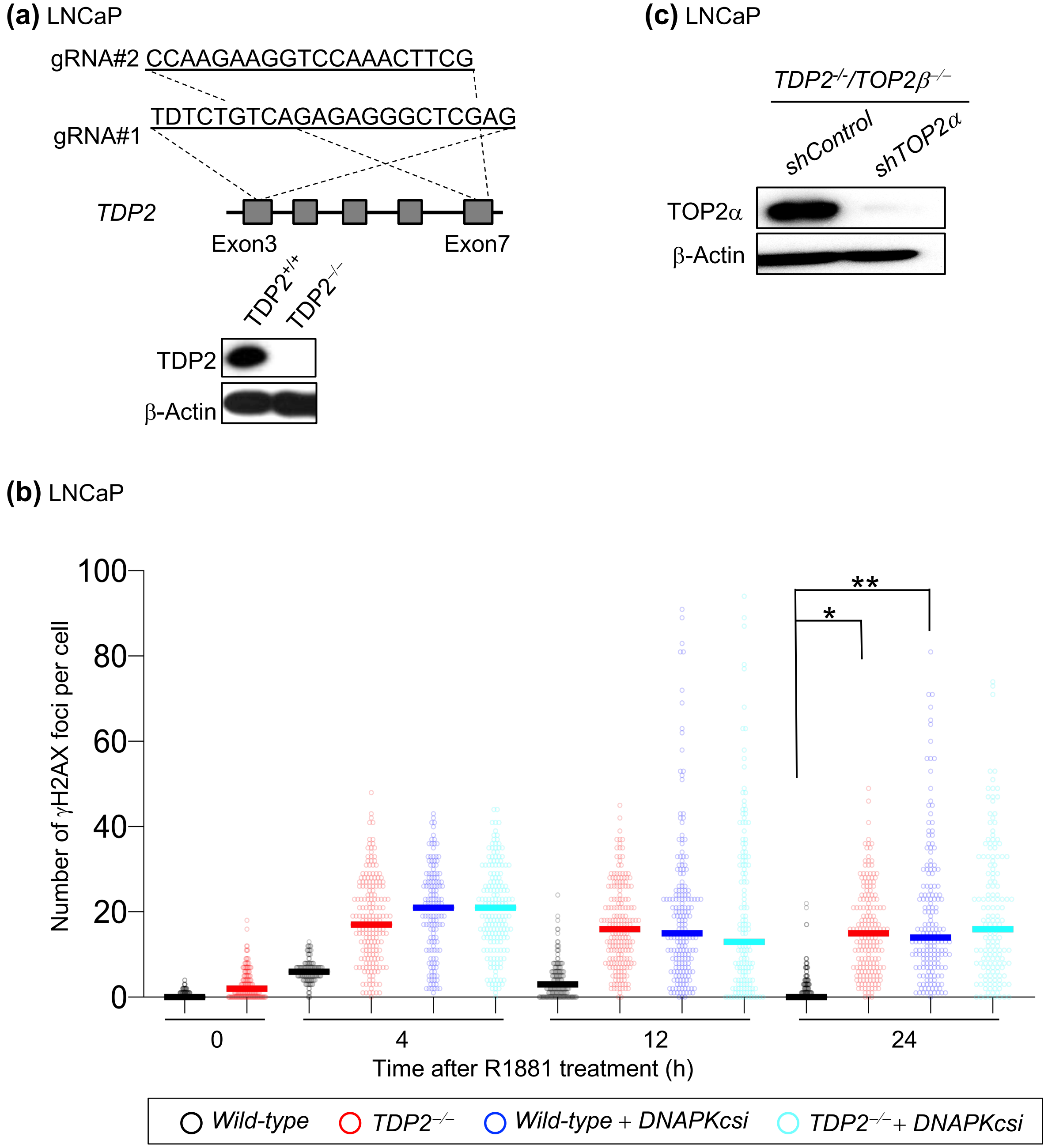
(a) LNCaP
gRNA#2 CCAAGAAGGTCCAAACTTCG
gRNA#1 TDTCTGTCAGAGAGGGCTCGAG
TDP2
Exon3
Exon7
TDP2+/+
TDP2−/−
TDP2
β-Actin
(b) LNCaP
Number of γH2AX foci per cell
100
80
60
40
20
0
**
*
0
4
12
24
Time after R1881 treatment (h)
Wild-type
TDP2−/−
Wild-type + DNAPKcsi
TDP2−/− + DNAPKcsi
(c) LNCaP
TDP2-/-/TOP2β-/-
shControl
shTOP2α
TOP2α
β-Actin

Rasel, *et al.*, Figure S3

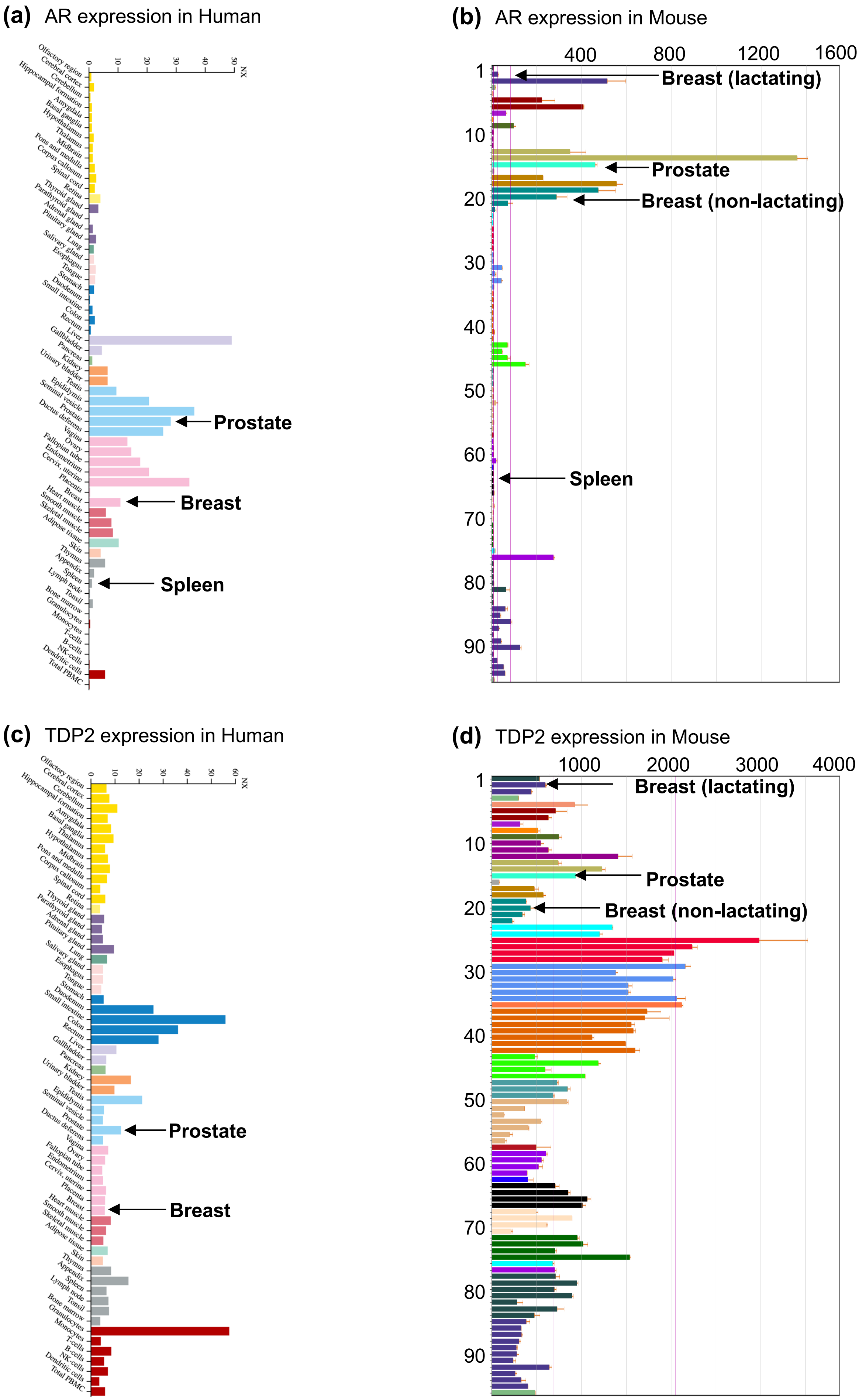

Rasel, *et al.*, Figure S4

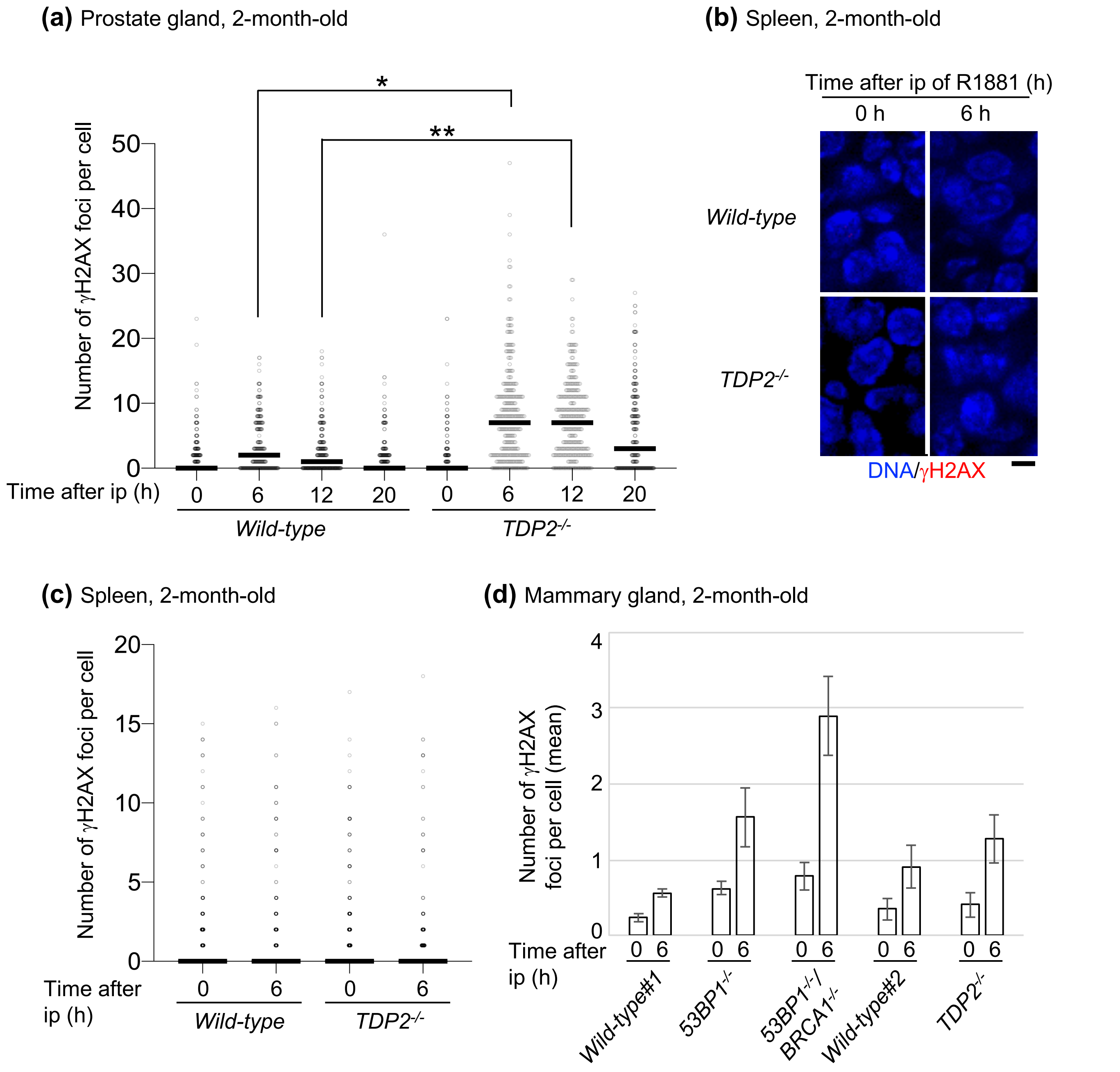

(a) Intestine and Spleen, 2-month-old

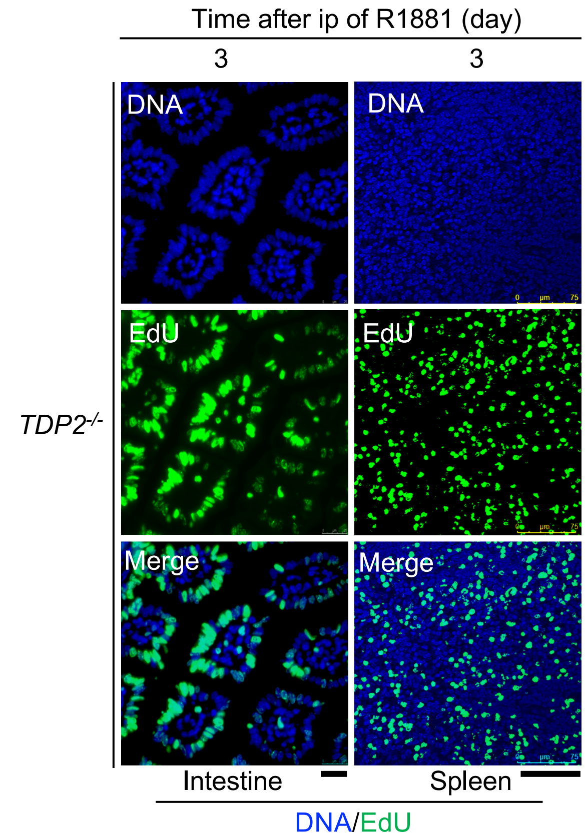

(b) Prostate gland, 2-month-old

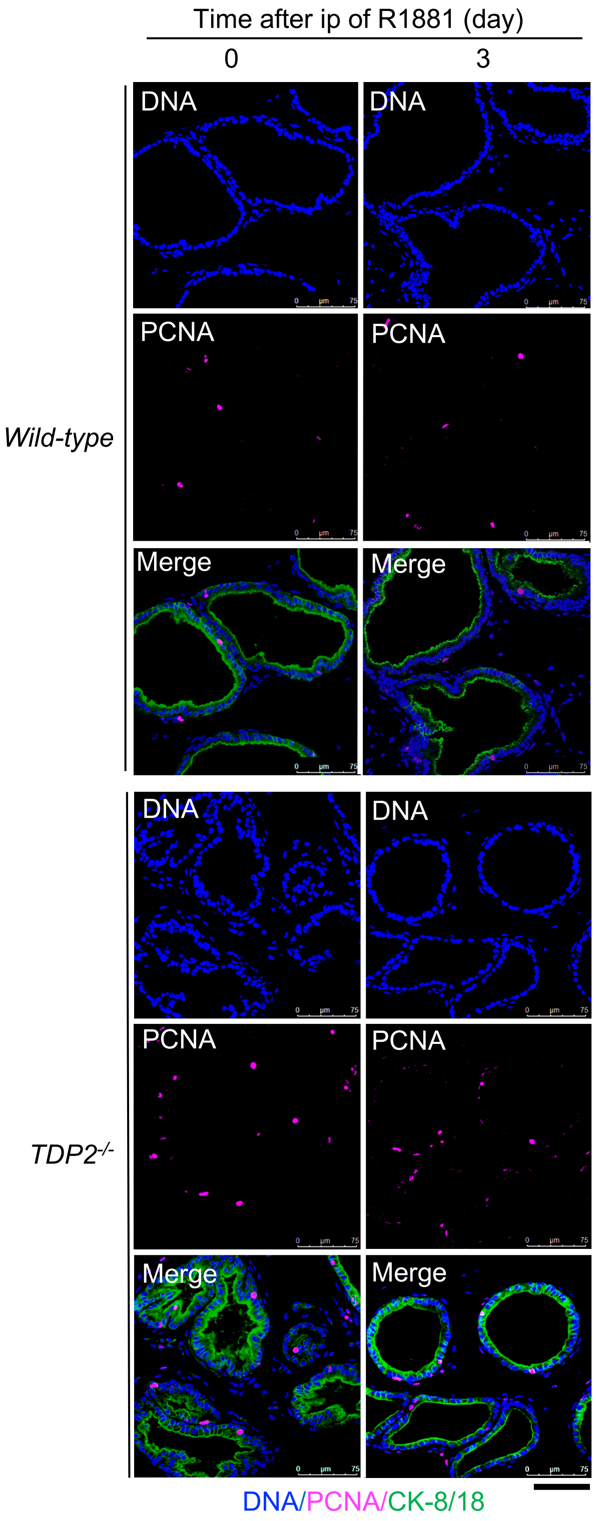

Supplement: Supplementary file 1 — Fig S1‐S5 [file GTC-25-450-s001.pdf]
