# Supplementary material for: TDP2 suppresses genomic instability induced by androgens in the epithelial cells of prostate glands
Source: Genes Cells. 2020 May 5;25(7):450–65. doi: 10.1111/gtc.12770 (PMC7497232; doi:10.1111/gtc.12770)
Supplement: Supplementary file 2 — Table S1‐S4 [file GTC-25-450-s002.pdf]

SUPPLEMENTAL TABLES

TABLE S1, List of Resources

**Reagent or Resource**	**Source**	**Identifier**
**Antibodies**		
Rat monoclonal α-Cytokeraitn-8/18	Developmental Studies Hybridoma Bank	Cat# TROMA-I, RRID:AB_531826
Goat polyclonal α-rabbit AlexaFluor 546	ThermoFisher	Cat# 11035, RRID: AB_2534093
Goat polyclonal α-rat AlexaFluor 488	Cell Signaling Technology	Cat# 4416S, RRID: AB_10693769
Mouse monoclonal α-γH2AX (clone JBW301)	Millipore	Cat# 05-636, RRID: AB_309864
Rabbit monoclonal α-γH2AX	Cell Signaling Technology	Cat# 9718, RRID: AB_2118009
Rabbit polyclonal α-CyclinA (clone C19)	Santa Cruz	Cat# sc-596, RRID: AB_631330
Mouse monoclonal α-Top2α	Novusbio	Cat# NBP2-47643
Mouse monoclonal α-Top2β	(Tsutsui et al., 2001)	N/A
Mouse monoclonal α-β-actin	Sigma	Cat# A5411
Goat polyclonal α-mouse AlexaFluor 488	ThermoFisher	Cat# A-11029, RRID: AB_2534088
Goat polyclonal α-rabbit AlexaFluor 488	ThermoFisher	Cat# A11034, RRID: AB_2576217
Goat polyclonal α-rabbit AlexaFluor 594	ThermoFisher	Cat# A11037, RRID: AB_2534095
Donkey polyclonal α-mouse HRP	Santa Cruz	Cat# sc-2314, RRID: AB_641170

**Chemicals, Peptides, and Recombinant Proteins**		
Charcoal/Dextran treated FBS	Hyclone Laboratories	Cat#SH30068.03
Giemsa's Stain Solution	Nacalai Tesque	Cat# 377114-35
Albumin, Bovine, F-V, pH5.2	Nacalai Tesque	Cat# 01863-48
Skim Milk for immunoassay	Nacalai Tesque	Cat# 31149-75
R1881	Sigma	Cat# R0908
Flutamide	Wako	Cat# 069-04851
NU7441(KU-57788)	Selleck	Cat# S2638
17β-Estradiol solution	Sigma	Cat# E-060
Hoechst 33258	Nacalai Tesque	Cat # 04928-92
Paraformaldehyde	Wako	Cat# 16320145
Lipofectamine 3000 Transfection Kit	ThermoFischer	Cat# L3000008
FuGENE HD Transfection Reagent	Promega	Cat# E2312
Doxycycline Hydrochloride	MP Biomedicals, Inc.	Cat# 195044
Fluoro-KEEPER Antifade Reagent	Nacalai Tesque	Cat# 12745-74
2.5 g/l-Trypsin/1 mmol/l-EDTA Solution	Nacalai Tesque	Cat# 35554-64
Propidium iodine (PI)	DOJINDO	Cat# P378
**Critical Commercial Assays**		
GeneArt Seamless Cloning Enzyme Mix	ThermoFisher	Cat# A14606
Click-iT® EdU Alexa Fluor® 488 Imaging Kits	Invitrogen	Cat# C10337
**Experimental Models: Cell Lines**		
Human: LNCaP, *wild-type*	ATCC	Cat# CRL-1740
Human: Lenti-X™ 293T	TAKARA	Cat# 632180
**Experimental Models: Organisms/Strains**		

The mutant genotypes of LNCaP are listed in Table S2	This work	
**Oligonucleotides**		
The primers are listed in Table S3	This work	
Mice: C57BL/6J		N/A
Mice: C57BL/6J *TDP2* $^{-/-}$		N/A
**Recombinant DNA**		
Plasmid: pSpCas9(BB)-2A-Puro(pX459)	(Ran, 2013)	Cat# 48139
Plasmid: pLKO.1	(Hoa et al., 2016)	Cat#8453

TABLE S2, List of Mutant Genotypes used in this study

Genotype	Name of cell line and species	Treat-ment[a]	Marker genes	Source	Identifier
TDP2 $^{-/-}$	Human LNCaP			This study	N/A
TOP2β $^{-/-}$	Human LNCaP				
TOP2β $^{-/-}$/ *TDP2* $^{-/-}$	Human LNCaP				

TABLE S3 List of oligonucleotides used in this study

Reagent or resource	Source	Identifier
Oligonucleotides (gRNA sequence)		
LNCaP, *TDP2,* gRNA#1 (for gene disruption),5'-TCTGTCAGAGAGGGCTCGAG -3'	Eurofin	N/A
LNCaP, *TDP2,* gRNA#2 (for gene disruption),5'-CGAAGTTTGGACCTTCTTGG -3'	Eurofin	N/A
LNCaP, *TOP2β,* gRNA (for gene disruption), 5'-CGGCGTGGGCGGCGGCAACG -3'	Eurofin	N/A
Oligonucleotides (shRNA sequences for pLKO.1 vector)		

TOP2α, forward primer, 5'-CCGGCCCATTGTAAAGGTATCTAAACTCGAGTTTAGATACCTTTACAATGGGTTTTT -3'	Eurofin	N/A
TOP2α, reverse primer, 5'-AATTAAAAACCCATTGTAAAGGTATCTAAACTCGAGTTTAGATACCTTTACAATGGG -3'	Eurofin	N/A

TABLE S4 Actual number of cells counted in each experiment

**Figure 1**			
**Figure**	**Genotype/ Condition**	**Experiment**	**Number of cell counted**
1c	*Wild-type* R1881 (-)	Exp. 01	73
		Exp. 02	67
		Exp. 03	59
	Wild-type R1881 (+)	Exp. 01	51
		Exp. 02	68
		Exp. 03	51
1e	*Wild-type* R1881 (-) Flutamide (-)	Exp. 01	73
		Exp. 02	67
		Exp. 03	59
	Wild-type R1881 (-) Flutamide (+)	Exp. 01	66
		Exp. 02	62
		Exp. 03	53
	Wild-type R1881 (+) Flutamide (-)	Exp. 01	51
		Exp. 02	68
		Exp. 03	51
	Wild-type R1881 (+) Flutamide (+)	Exp. 01	55
		Exp. 02	77
		Exp. 03	51
1g	*Wild-type* R1881 (-)	Exp. 01	73
		Exp. 02	67
		Exp. 03	59
	Wild-type R1881 (+)	Exp. 01	51
		Exp. 02	68
		Exp. 03	51
	TOP2β -/-R1881 (-)	Exp. 01	58
		Exp. 02	61
		Exp. 03	74
	TOP2β -/- R1881 (+)	Exp. 01	72

		Exp. 02	78
		Exp. 03	54
	TOP2β -/-/shControl R1881 (-)	Exp. 01	74
		Exp. 02	74
		Exp. 03	72
	TOP2β -/-/shControl R1881 (+)	Exp. 01	62
		Exp. 02	71
		Exp. 03	68
	TOP2β -/-/shTop2α R1881 (+)	Exp. 01	65
		Exp. 02	61
		Exp. 03	59
	TOP2β -/-/shTop2α R1881 (+)	Exp. 01	53
		Exp. 02	52
		Exp. 03	57

Figure 2

**Figure**	**Genotype/ Condition/ Time**	**Experiment**	**Number of cell counted**
2c	*Wild-type* 0 h	Exp. 01	73
		Exp. 02	67
		Exp. 03	59
	Wild-type 4 h	Exp. 01	51
		Exp. 02	68
		Exp. 03	51
	Wild-type 12 h	Exp. 01	51
		Exp. 02	56
		Exp. 03	76
	Wild-type 24 h	Exp. 01	65
		Exp. 02	79
		Exp. 03	69
	TDP2 -/- 0 h	Exp. 01	64
		Exp. 02	54
		Exp. 03	60
	TDP2 -/- 4 h	Exp. 01	69
		Exp. 02	66
		Exp. 03	64
	TDP2 -/- 12 h	Exp. 01	74
		Exp. 02	79
		Exp. 03	52
	TDP2 -/- 24 h	Exp. 01	71

		Exp. 02	64
		Exp. 03	61
	Wild-type + DNAPKcsi 4 h	Exp. 01	51
		Exp. 02	54
		Exp. 03	53
	Wild-type + *DNAPKcsi* 12 h	Exp. 01	65
		Exp. 02	57
		Exp. 03	70
	Wild-type + *DNAPKcsi* 24 h	Exp. 01	63
		Exp. 02	65
		Exp. 03	56
	TDP2 -/- + *DNAPKcsi* 4 h	Exp. 01	59
		Exp. 02	62
		Exp. 03	69
	TDP2 -/- + *DNAPKcsi* 12 h	Exp. 01	52
		Exp. 02	51
		Exp. 03	51
	TDP2 -/- + *DNAPKcsi* 24 h	Exp. 01	55
		Exp. 02	53
		Exp. 03	53
2e	*TDP2 -/-* 0 h	Exp. 01	64
		Exp. 02	54
		Exp. 03	60
	TDP2 -/- 4 h	Exp. 01	69
		Exp. 02	66
		Exp. 03	64
	$TDP2^{-/-}/TOP2\beta^{-/-}/shControl$ 0 h	Exp. 01	58
		Exp. 02	55
		Exp. 03	57
	$TDP2^{-/-}/TOP2\beta^{-/-}/shControl$ 4 h	Exp. 01	52
		Exp. 02	61
		Exp. 03	62
	$TDP2^{-/-}/TOP2\beta^{-/-}/shTOP2\alpha$ 0 h	Exp. 01	63
		Exp. 02	62
		Exp. 03	54
	$TDP2^{-/-}/TOP2\beta^{-/-}/shTOP2\alpha$ 4 h	Exp. 01	58
		Exp. 02	61
		Exp. 03	58
2f	*Wild-type* R1881 (-)	Exp. 01	50

		Exp. 02	50
		Exp. 03	50
	Wild-type R1881 (+)	Exp. 01	50
		Exp. 02	50
		Exp. 03	50
	TDP2-/- R1881 (-)	Exp. 01	50
		Exp. 02	50
		Exp. 03	50
	TDP2-/- R1881 (+)	Exp. 01	50
		Exp. 02	50
		Exp. 03	50
**Figure 3**			
**Figure**	**Genotype/ Condition/ Time**	**Experiment**	**Number of cell counted**
3b	*Wild-type* 0 h	Exp. 01	121
		Exp. 02	100
		Exp. 03	100
	Wild-type 6 h	Exp. 01	109
		Exp. 02	100
		Exp. 03	100
	Wild-type 12 h	Exp. 01	100
		Exp. 02	100
		Exp. 03	100
	Wild-type 20 h	Exp. 01	122
		Exp. 02	100
		Exp. 03	100
	TDP2 -/- 0 h	Exp. 01	101
		Exp. 02	100
		Exp. 03	100
	TDP2 -/- 6 h	Exp. 01	110
		Exp. 02	100
		Exp. 03	100
	TDP2 -/- 12 h	Exp. 01	107
		Exp. 02	100
		Exp. 03	100
	TDP2 -/- 20 h	Exp. 01	103
		Exp. 02	100
		Exp. 03	100
3d	*Wild-type#1* 6 h	Exp. 01	151

		Exp. 02	76
		Exp. 03	146
	53BP1 -/- 6 h	Exp. 01	220
		Exp. 02	215
		Exp. 03	217
	53BP1$^{-/-}$/BRCA1$^{-/-}$ 6 h	Exp. 01	186
		Exp. 02	297
		Exp. 03	124
	Wild-type#2 6 h	Exp. 01	118
		Exp. 02	103
		Exp. 03	164
	TDP2 -/- 6 h	Exp. 01	130
		Exp. 02	115
		Exp. 03	115
**Figure 4**			
**Figure**	**Genotype/ Condition/ Time**	**Experiment**	**Number of cell counted**
4c	*Wild-type* day 0	Exp. 01	752
		Exp. 02	714
		Exp. 03	733
	Wild-type day 3	Exp. 01	795
		Exp. 02	728
		Exp. 03	768
	TDP2 -/- day 0	Exp. 01	661
		Exp. 02	564
		Exp. 03	705
	TDP2 -/- day 3	Exp. 01	616
		Exp. 02	675
		Exp. 03	882
4d	*Wild-type* day 0	Exp. 01	650
		Exp. 02	661
		Exp. 03	880
	Wild-type day 3	Exp. 01	710
		Exp. 02	872
		Exp. 03	864
	TDP2 -/- day 0	Exp. 01	576
		Exp. 02	613
		Exp. 03	545
	TDP2 -/- day 3	Exp. 01	751

		Exp. 02	851
		Exp. 03	720
**Figure 5**			
**Figure**	**Genotype/ Condition/ Time**	**Experiment**	**Number of cell counted**
5b	*Wild-type* 2 m	Exp. 01	650
		Exp. 02	661
		Exp. 03	880
	Wild-type 6 m	Exp. 01	607
		Exp. 02	783
		Exp. 03	682
		Exp. 04	650
		Exp. 05	534
	TDP2 -/- 2 m	Exp. 01	576
		Exp. 02	613
		Exp. 03	545
	TDP2 -/- 6 m	Exp. 01	877
		Exp. 02	653
		Exp. 03	532
		Exp. 04	926
		Exp. 05	755
5d	*Wild-type* 2 m	Exp. 01	537
		Exp. 02	789
		Exp. 03	642
	Wild-type 6 m	Exp. 01	687
		Exp. 02	623
		Exp. 03	691
		Exp. 04	795
		Exp. 05	662
	TDP2 -/- 2 m	Exp. 01	799
		Exp. 02	1249
		Exp. 03	1147
	TDP2 -/- 6 m	Exp. 01	2267
		Exp. 02	1909
		Exp. 03	1742
		Exp. 04	2594
		Exp. 05	2463

SUPPLEMENTARY FIGURE LEGENDS

FIGURE S1 (related to Figure 1)

Cell cycle analysis, generation of *TOP2β*$^{-/-}$ and *TOP2β*$^{-/-}$*/shTOP2α* LNCaP cells.

(a) The cell cycle analysis. The cells were stained with FITC-conjugated anti-BrdU antibody to measure BrdU incorporation into genomic DNA and with propidium iodide (PI) to measure the total DNA. *Wild-type* LNCaP cells were analysed before (cyclin) and after 48-h of serum starvation. (b) Scatter dot plot representing the distribution of number of γH2AX foci in *wild-type* LNCaP cell (related to Figure 1c). The lines on the data represent median values. A Single asterisk indicates $p < 0.0001$, which was calculated by an unpaired two-tailed *t*-test in the GraphPad Prism. (c) Scatter dot plot representing the distribution of number of γH2AX foci in *wild-type* LNCaP cell (related to Figure 1e). The lines on the data represent median values. A Single asterisk indicates $p < 0.0001$, which was calculated by an unpaired two-tailed *t*-test. (d) Schematic diagram of *TOP2β*$^{-/-}$ genomic locus, target location, and gRNA sequence. "ATG" indicates the start codon of the TOP2β gene (top). Targeting events were confirmed by western blotting analysis (lower) of TOP2β. Blot shows *wild-type* and TOP2*β*$^{-/-}$ cells, generated for this study. β-Actin was a loading control. (e) Western blot analysis of Top2α expression at 0 and 48 h after serum starvation in *wild-type* LNCaP cells. β-Actin was a loading control. (f) Western blot analysis of TOP2α-shRNA-treated (shTOP2α) LNCaP cells. Lanes 1, 2, and 3 show *wild-type*, *TOP2β*$^{-/-}$, and control-shRNA-treated cells, respectively, representing positive controls. Lane 4 shows a successful depletion of TOP2α by shRNA. β-Actin was a loading control. (g) Scatter dot plot representing the distribution of number of γH2AX foci in the indicated genotypes of LNCaP cell (related to Figure 1g). The lines on the data represent median values. Single, double, and triple asterisks indicate $p = 9.4 \times 10^{-1}$ (no significant difference), $p < 0.0001$, and $p = 5.0 \times 10^{-1}$ (no significant difference), respectively, which were calculated by an unpaired two-tailed *t*-test.

FIGURE S2 (related to Figure 2)

Generation of *TDP2*$^{-/-}$ LNCaP cells and quantification of R1881-induced γH2AX foci.

(a) Schematic diagram of *TDP2* genomic locus, target location, and gRNA sequence. Western blot analysis of TDP2. Western blot shows *wild-type* and TDP2$^{-/-}$ cells, generated for this study. *β*-Actin was a loading control. (b) Scatter dot plot representing the distribution of number of γH2AX foci at 0, 4, 12, and 24 h in the indicated genotypes of LNCaP cell (related

to Figure 2c). The lines on the data represent median values. Both single, and double asterisks indicate p <0.0001, which were calculated by an unpaired two-tailed t-test in the GraphPad Prism. (c) Western blot analysis of *TOP2α*-shRNA-treated (shTOP2α) LNCaP cells. Blot shows control-shRNA-treated cells, representing positive control and a successful depletion of TOP2α by shRNA. β-Actin was a loading control.

FIGURE S3

Tissue-specific expression of androgen receptor and TDP2.

(a) Overview of Tissue-specific androgen receptor (AR) RNA expression in Human. Consensus Normalized eXpression (NX) levels for 55 tissue types and 6 blood cell types, created by combining the data from the three transcriptomics datasets (HPA, GTEx and FANTOM5) using the internal normalization pipeline (proteinatlas.org/ENSG00000169083-AR/tissue). (b) AR expression in different tissues/cells in mouse (http://biogps.org/#goto=genereport&id=11835): 1. MEF, 2. Mammary_gland_lact, 3. Adipose_white, 4. Lung, 5. Placenta, 6. Uterus, 7. Ovary, 8. Bladder, 9. Umbilical_cord, 10, Epidermis, 11. Stomach, 12. Intestine_large, 13. Intestine_small, 14. Salivary gland, 15. Lacrimal_gland, 16. Prostate, 17. Pancreas, 18. Pituitary, 19. Adrenal gland, 20. Skeletal muscle, 21. Mammary gland, non-lactating, 22. Adipose_brown, 23. Heart, 24. Embryonic stem line_V26_2 pl6, 25. Embryonic stem line Brucc4 pi3, 26. Stem cells HSC, 27. Common myeloid progenitor, 28. Granulo mono progenitor, 29. Mega erythrocyte progenitor, 30. Thymocyte DP CD4+CD8+, 31. Thymocyte SP CD4+, 32. Thymocyte SP CD8+, 33. T-cells CD4+, 34. T-cells CD8+, 35. T-cells foxP3+, 36. NK cells, 37. B-cells GL7 negative Alum, 38. B-cells GL7 positive KLH, 39. B-cells GL7 positive Alum, 40. B-cells GL7 negative KLH, 41. Follicular B-cells, 42. B-cells marginal zone, 43. Baf3, 44. Mast cells, 45. Mast cells IgE, 46. Mast cells IgE+antigen 1hr, 47. Mast cells IgE+antigen 6hr, 48. Dendritic cells myeloid CD8a-, 49. Dendritic cells lymphoid CD8a+, 50. Dendritic plasmacytold B220+, 51. Macrophage bone marrow 0hr, 52. Macrophage bone marrow 2hr LPS, 53. Macrophage bone marrow 6hr LPS, 54. Macrophage bone marrow 24h LPS, 55. Macrophage peri LPS thio 0hrs, 56. Macrophage peri LPS thio 1 hrs, 57. Macrophage peri LPS thio 7hrs, 58. Granulocytes macl+grl+, 59. Osteoclasts, 60. Osteoblast day 5, 61. Osteoblast day 14, 62. Osteoblast day 21, 63. Microglia, 64. Spleen, 65. Lymph nodes, 66. Bone marrow, 67. Bone, 68. Nih 3T3, 69. C2C12, 70. C3H10T1_2, 71. 3T3-L1, 72. MIMCD-3, 73. Neuro2a, 74. RAW 264 7, 75. Mln6, 76. Liver, 77. Kidney, 78. Retinal pigment epithelium, 79. Retina, 80. Iris, 81. Ciliary bodies,

82. Eyecup, 83. Lens, 84. Cornea, 85. Cerebral cortex prefrontal, 86. Cerebral cortex, 87. Amygdala, 88. Hippocampus, 89. Olfactory_bulb, 90. Spinal cord, 91. Hypothalamus, 92. Cerebellum, 93. Nucleus_accumbens, 94. Dorsal_striatam, 95. Dorsal_root_ganglia, and 96. Testis. High-throughput gene expression profiling from a diverse array of normal tissues, organs, and cell lines in mice. (c) Overview of tissue-specific TDP2 RNA expression in Human. Consensus Normalized eXpression (NX) levels for 55 tissue types and 6 blood cell types, created by combining the data from the three transcriptomics datasets (HPA, GTEx and FANTOM5) using the internal normalization pipeline (https://www.proteinatlas.org/ENSG00000111802-TDP2/tissue). (d) TDP2 expression in different tissues/cells in mouse (http://biogps.org/#goto=genereport&id=56196) as in (b). High-throughput gene expression profiling from a diverse array of normal tissues, organs, and cell lines in mice.

FIGURE S4 (related to Figure 3)

R1881-induced γH2AX foci in the prostate gland and spleen

(a) Scatter dot plot representing the distribution of number of γH2AX foci in the prostate at 0, 6, 12, and 20 h in the indicated genotypes of mice (related to Figure 3b). The lines on the data represent median values. Both single, and double asterisks indicate p <0.0001, which were calculated by an unpaired two-tailed t-test. (b) Representative images of the R1881-induced γH2AX foci in the spleen. Scale bar represents 25 μm. (c) Scatter dot plot representing the distribution of number of γH2AX foci in the spleen at 0 and 6 h in the indicated genotypes of mice. The lines on the data represent median values. (d) The average number of E2-induced γH2AX foci in the epithelial cells of the mammary gland in the indicated genotypes. The data of *wild-type* #1, *53BP1*$^{-/-}$, and *53BP1*$^{-/-}$/*BRCA1*$^{-/-}$ mice were re-calculated from the data that has been previously published (Sasanuma et al., 2018).

FIGURE S5 (related to Figure 4)

R1881-induced EdU^{+} or PCNA^{+} epithelial cells in the tissues.

(a) Representative images of EdU labelled (green) Intestine and Spleen as positive control after 3-day continuous ip with EdU and/or R1881. Green labelling indicates EdU^{+} cells which actively incorporate EdU during replication. (b) Representative images of PCNA^{+} luminal epithelial cells in the prostate gland. The magenta and green labelling represent PCNA and

CK-8/18, respectively. CK-8/18 is the marker of prostate luminal epithelial cells. Scale bar represents 25 μm and 75 μm for Intestine and Spleen, respectively.
